# Changes in water and land: the reconstructed Viennese riverscape from 1500 to the present

**DOI:** 10.1007/s12685-013-0074-2

**Published:** 2013-07-03

**Authors:** Severin Hohensinner, Bernhard Lager, Christoph Sonnlechner, Gertrud Haidvogl, Sylvia Gierlinger, Martin Schmid, Fridolin Krausmann, Verena Winiwarter

**Affiliations:** 1 Institute of Hydrobiology and Aquatic Ecosystem Management (IHG), University of Natural Resources and Life Sciences Vienna (BOKU), Max-Emanuel-Str. 17, 1180 Vienna, Austria; 2 Municipal and Provincial Archives of Vienna, Rathaus, 1082 Vienna, Austria; 3 Centre for Environmental History (ZUG), Alpen-Adria University Klagenfurt, Schottenfeldgasse 29, 1070 Vienna, Austria; 4 Institute of Social Ecology (IFF, ZUG), Alpen-Adria University Klagenfurt, Vienna, Austria

**Keywords:** River morphology, River regulation, Fluvial dynamics, Danube, Vienna

## Abstract

Medieval Vienna was situated at the main arm of the swiftly flowing alpine Danube. From the fourteenth century onwards, the river gradually moved away from the city. This marked the beginning of 500 years of human intervention to prevent further displacement of the river and to preserve the waterway as a vital supply line. Archival research and the GIS-based reconstruction of the past riverscape allow a new view about the co-evolution of the city and the river. Following major channel changes in 1565/1566, repeated attempts to force the main arm into the old river bed were undertaken. By the early seventeenth century, the Viennese had accepted the new situation. Resources were now spent on maintaining the waterway to the city via the remaining *Wiener arm*. After the second Ottoman siege in 1683, improving the navigability of the *Wiener arm*, in conjunction with major expansions of the fortifications, became the main issue. Between 1775 and 1792, the first systematic, effective flood protection measures were established. These substantially influenced fluvial dynamics and enabled urban development in parts of the former floodplain. The all-embracing transformation of the dynamic riverscape into stabilised areas enabling urban growth and secure waterways was not achieved until 1875. With this successful “re-invention” of the Viennese Danube, an irreversible path was struck in the common life of the city and the river, a path which is still decisive for the interaction of Vienna with that great European river.

## Introduction

Among the larger medieval cities, Vienna stands out as having been situated at the main arm of a swiftly flowing alpine river, rather than near the river mouth or on the coast. The Danube branched out into a huge floodplain to the north of the city. In the fourteenth century, documentary evidence suggests that the Danube was gradually moving away from the historic city centre (Thiel 1904; Krcmar 1924; Altfahrt 2000). From then on, the Viennese authorities intervened for over 500 years to prevent further displacement of the river and to preserve the waterway as a vital supply line for the city.

We trace the natural and human-induced transformation processes of the Viennese Danube riverscape from the late fifteenth century to the present. In a radical departure from usual historical accounts, we tell the story centred on the river’s agency in prompting human reaction to its changes. GIS reconstructions of the riverscape at 11 points in time form the basis of the study (1529, 1570, 1632, 1663, 1726, 1780, 1817, 1849, 1875, 1912 and 2010; Lager 2012). A detailed description of the underlying historical sources and the methodology is given in this issue (Hohensinner et al. 2013). Several historical and cartographic studies from the nineteenth and twentieth centuries provided a basis for our research. The earliest among them is the work of Florian Pasetti Ritter von Friedenburg, a member of the *Danube Regulation Commission* between 1850 and 1868, who provides a wealth of information about the state of the Danube riverscape and of previous hydraulic constructions (Pasetti et al. 1850; Pasetti 1859, 1862). Thiel (1904, 1906), Slezak (1977, 1978, 1980) offer in-depth information about early river engineering measures from the sixteenth to the eighteenth century. With regard to historical plans and maps of Vienna and the Danube, the “Donauatlas*”* from Mohilla and Michlmayr (1996) is also of fundamental importance in this context.

Historical changes are well documented for the late nineteenth century onwards. In this article, we focus on the earlier periods, about which little is known so far, and we trace the transformation of the Viennese Danube during six phases. While the first phases prior to 1683 can be characterised as a half-hearted fight against the inevitable, regulation efforts increased over time and culminated in the main regulation of 1870–1875. Considered definitive by contemporaries, it has nevertheless proven to be only temporary. The river keeps changing, regulation work continues, and new threats characterise the twentieth and twenty first centuries.

Over the past 500 years, the intensity of human intervention increased and so did the associated impacts on the biophysical riverine environment of Vienna. We can show that from the sixteenth century onwards, the Viennese Danube was a socio-natural site, shaped by the practices of humans and shaping these practices by its hybrid socio-natural dynamic (Winiwarter and Schmid 2008).

## The Viennese river landscape

The Danube provided timber from the riparian woods, fishes from the different water bodies, pasture in the floodplain and waterborne transportation. The latter was vital for the provisioning of the city but also precarious because of the river’s tendency to move away from the city. Maintaining the waterway close to the city was a major task for the Viennese municipal authorities and the various imperial institutions (Sonnlechner et al. 2013, in this issue). Historically, Vienna was located for at least 800 years not directly at the main stem of the Danube (Thiel 1904; Klusacek and Stimmer 1995) but on top of an older and higher Pleistocene river terrace (Fink and Majdan 1954; Lisiecki and Raymo 2005). The border between this *Stadtterrasse* and the recent, post-glacial alluvium of the Danube formed up to 12,000 years ago coincides closely with the outer fortification of the Roman legionary camp *Vindobona* and approximately with the medieval city walls (Suess 1862; Brix 1970). Therefore it is generally assumed that a main branch of the Danube extended to the city during roman times and the early to high Middle Ages (Opll 1986; Grupe and Jawecki 2004).

Beginning at the latest in the 12th century, in a first phase of channel shifting, the Danube moved away from the town to the north, which enabled the expansion of the urban area into the alluvial riverscape (Weschel 1824; Buchmann et al. 1984; Altfahrt 2000). Up to the early fifteenth century, two such areas in the so-called *Oberer Werd* (corresponding approximately to today’s *Rossau* in Vienna’s 9th district) and *Unterer Werd* (*Leopoldstadt*, 2nd district) had already been settled, partly on formerly water-covered areas close to the city (Haidvogl et al. 2013, in this issue).

Around 1500, the Danube was not pristine; it must already have shown the effects of human influence, mainly due to the use of riparian forests and probably also due to large-scale land use change in the upper catchment. As far as we know, hydraulic constructions were most probably restricted to the *Wiener arm*, a side arm that ran close to the city centre (Mitis 1835; Thiel 1904). The Danube riverscape consisted of numerous larger and smaller arms that were branched by several islands; some islands were several kilometres long. While the main arms featured extensive gravel bars, the smaller arms were able to develop differently, being straight, sinuous or even meandering (“anabranching river type”, Nanson and Knighton 1996). The river arms moved laterally in the riverscape over a distance of 6 km. This fostered regular erosion and accretion processes (Hohensinner and Jungwirth 2009; for more detail on Danube river morphology see Hohensinner et al. 2013, in this issue). Floods, small and large, were a regular feature of the Danube. Vienna’s younger settlements on the floodplain outside the city walls were particularly threatened by floods. Ice jam floods were a greater challenge than the summer floods after heavy rainfalls and thaw floods in spring. They were a typical phenomenon along the Viennese Danube, because the numerous branches of the river favoured the formation of jams (Pasetti 1859; k.k. HZB 1908). These could severely damage the populated areas of Vienna. For the reconstruction of historical states of the riverscape we documented the floods in a database, as their influence on the course of the river branches but also on measures humans would take to curb them are important for the co-evolutionary development of the city and the river (Sartori 1830; Suess 1862; Trimmel 1970).

## Wrestling with the river: a chronological overview

To follow the overview, the map of 1912 (Fig. 6) is a good starting point, as it links the current situation with the past. We highlighted landmarks in all reconstructions that allow to trace particular features through a landscape that is highly dynamic.

### Fighting against the inevitable (c. 1500–1610)

**Fig. 1:**
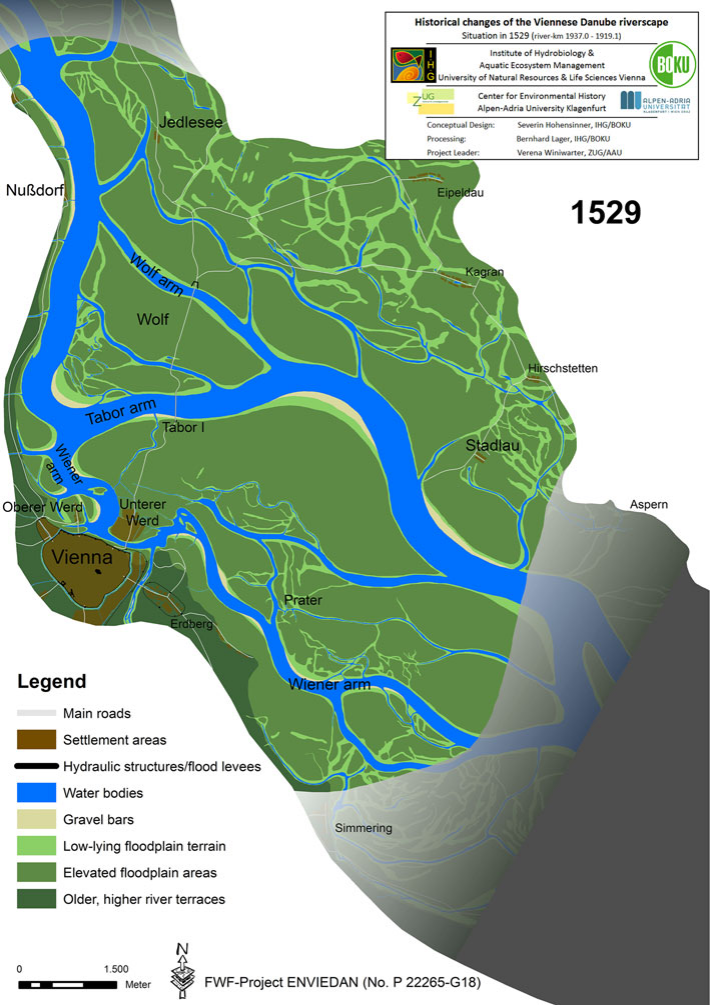
Reconstructed state of the Danube riverscape in Vienna in 1529

We have scattered evidence of river movement prior to the first reconstruction of 1529. In 1455, the main arm of the Danube can be located approximately 3.5 km north from the city. At that time, the hydraulic engineer Kaspar Hartneid was commissioned by the Viennese council upon advice of Sigismund, ruler of Tyrol and Archduke of Austria. Hartneid should maintain the flow in the vital waterway to the city, but he failed.^1^ Figure 1 shows the reconstructed situation of 1529 for which we used the “Meldeman-Plan” from 1529/30 as the main source.^2^ Though direct georeferencing in GIS is not possible, the plan provides valuable information on riverine structures. In combination with the situation in 1570, we could draw conclusions on the fluvial processes and the state in 1529 (compare Fig. 1 and the discussion of the plan in this issue, Hohensinner et al. 2013).

The situation in 1529 is fundamentally different from that of later centuries, the main arm (*Tabor arm*, named after the fortified toll building called *Tabor* at the *Tabor bridge*) showing a sinuous course that stretched far south towards the city (Fig. 1). A side arm, later called *Wiener arm* and the predecessor of today’s *Donaukanal*, formed the precarious supply line between the main channel and the city. In 1529, the bifurcation of the two arms lay approximately 1.6 km north of the city. By 1570, this distance had shrunk to c. 1.3 km, indicating a constant shift of the main branch towards the city from at least 1455 onwards over more than 100 years. The reason for this lies in the gradual expansion of a distinct river bend of the main branch, which thereby moved closer to the city.^3^ However, this process did not mitigate the ongoing problem of the aggrading *Wiener arm*, as documented by several sources (Thiel 1904).

In 1529, the *Wolf*, the floodplain within the river bend of the main arm, was rather compact compared to its state in 1570. The *Wolf* must have constituted a morphologically stable area of the riverscape at least until 1547. Given the bridge lengths described by Wolfgang Schmeltzl (1548), only two noteworthy river arms existed. According to Thiel (1904), the first constructions were implemented probably around 1540 upstream from the *Wolf* on the left bank opposite the village of Nußdorf (Fig. 1).^4^ Whether these constructions were meant to prevent the evolution of new side arms towards the village Jedlesee or towards the *Wolf* remains unclear (Fig. 1). Regulation efforts were boosted at that site between 1548 and 1554/58, which may indicate increased fluvial dynamics upstream from Vienna. The legal and financial responsibility for hydraulic works in the sixteenth and seventeenth centuries was, besides the municipal authorities, on the government and treasury of Lower Austria, the court treasury (*Hofkammer*) and the court’s council of war (*Hofkriegsrat*). For a major hydro-engineering project, both the city and the Habsburg ruler had to negotiate with these institutions and with various other private and official stakeholders, including among others shipmasters, fishermen, bridge-masters and fortification engineers. That made the realisation of such measures a complex task (for details see Sonnlechner et al. 2013, in this issue).^5^

**Fig. 2:**
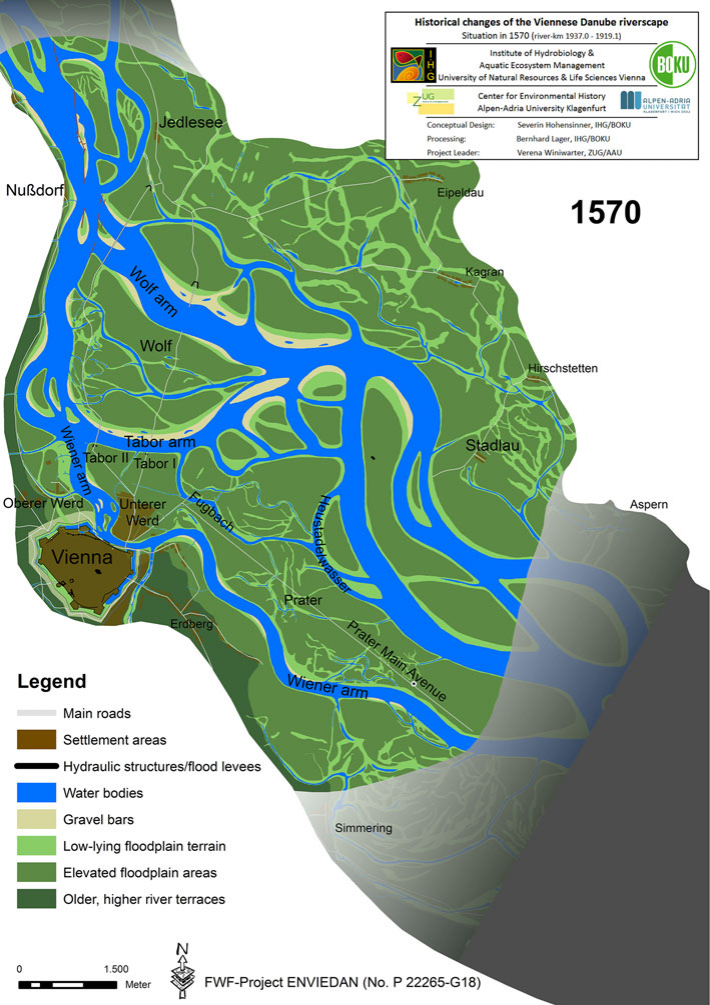
Reconstructed state of the Danube riverscape in Vienna in 1570. The former main arm (*Tabor arm*) showed already significant sedimentation while the new main arm (*Wolf arm*) increased in width. *Tabor* I (until 1565) and *Tabor* II are indicated together with the bridges

By 1565, the river, redirecting the flow towards the north, had formed new arms close to Nußdorf and substantially widened the *Wolf arm*.^6^ Although regulation measures near Nußdorf were implemented to prevent a major relocation of the Danube into the widened *Wolf arm*, this shift could not be halted: during the ice jam flood in the spring of 1565, the impact of which was aggravated by the very high summer flood in 1566, the Danube finally cut off its extensive river bend and shifted its main branch away from the city to the *Wolf arm*.^7^ Some years before, between 1547 and 1565, the Danube had already cut off the vertex of its river bend close to the city and shifted the new vertex more than 1 km towards the east (compare Figs. 1, 2). This channel change is likely to have considerably altered flow conditions in the *Wiener arm* towards the city that, in turn, triggered erosion and accretion processes: the *Obere Werd* (today’s *Rossau*) gained considerable new aggraded terrain, while the *Untere Werd* (*Leopoldstadt*) lost land in some places but gained in others. Figure 2 illustrates the result of these far-reaching morphological changes in 1570.^8^ They are fundamental to understanding the continuous regulation efforts in the centuries that followed and comprehending how exactly the provisioning of the city had become a critical issue.

But what were the driving forces behind such a major rearrangement of the Danube riverscape? Over the long term, tectonic subsidence has played a major role. The study site is located in the *Viennese Basin* between the *Wiener Pforte Gap* (a short breakthrough section upstream from Nußdorf) and the *Schwechat Tief* where the geological basement has subsided more than 5,000 m within the last 17 million years, a process that still continues (Grupe and Jawecki 2004). This geological process is a generally accepted explanation for the tendency of the Viennese Danube to shift towards the northeast since Roman times (Keiler and Thaller 2009). The second factor can be potentially found in large-scale medieval forest clearings in the Austria Danube catchment, leading to an all-time high in assarted and agriculturally used land around the mid-fourteenth century (Sonnlechner 2000; Csendes and Opll 2001). Soil erosion must have resulted in an enormous loss of land; tributaries introduced high amounts of bedload into the Danube which boosted fluvial dynamics (Kern 1994; Bork et al. 1998). In addition, according to the sources found and to literature, the years between 1565 and 1571 in particular seem to have been outstanding in terms of regular ice jams and heavy floods that caused severe damage (Pfister 1999; Glaser 2008).^9^ This period corresponds to the *Grindelwald Fluctuation*, the first extreme phase of the *Little Ice Age* lasting from the 1560s to the 1620s (Pfister 1980, 2007; Behringer 1999).

Assessing the direct influence of climate change on the local fluvial dynamics in the sixteenth century is difficult, but embedding the findings into the larger frame of a central European climate history helps to interpret the dramatic changes in the Viennese riverscape. Besides the large-scale framework conditions, the local situation, i.e. the morphological development stage of the respective river section, is also of fundamental importance. The river’s ability to develop a sinuous or even meandering course is a function of the given flow regime, channel slope, sediment supply, etc.; river patterns can only develop within a certain range (Howard 1988). When a threshold inherent to the river type is reached or even exceeded, the river’s morphology will change. In the case of the Viennese Danube, first indications of an upcoming major channel change are reported upstream from Nußdorf in the 1550s, when the right (southern) bank was eroded.^10^ This led to a redirection of the main current in the downstream section near Nußdorf from south (*Tabor arm*) to southeast towards the *Wolf arm* (compare Figs. 1, 2). As a result, the highly sinuous *Tabor arm* was naturally cut off and the river formed a new and straighter channel, the *Wolf arm*. Tectonic subsidence may have generally promoted such a development over the long term, but the historical sources and the GIS-reconstruction of the river dynamics highlight the special role of a river’s morphological development stage and that of climatic changes. In the case of Vienna, the cold phase of the *Grindelwald Fluctuations* may be supposed to have boosted an already forthcoming rearrangement of the riverscape.

After 1565/66, the former main arm (*Tabor arm*) showed extensive terrestrialization, as indicated by large gravel bars and newly vegetated areas at its inner bank. Once the *Wolf arm* had become the new main arm, it widened its channel profile up to c. 800 m in 1570.^11^ All the larger Danube arms combined added up to a total width of almost 1,300 m, which was bound to be unstable under the hydrological regime of the Danube.^12^ Under such conditions, a river typically starts to develop a smaller but more sinuous channel within the wider river bed. Over time the channel width decreases, while the sinuosity increases. The *Wolf arm* experienced such a channel transformation after 1571, which we will discuss in the following chapter.

The fundamentally changed Danube arms, the repeated destruction of bridges by ice jams and substantial erosion of floodplain terrain necessitated a relocation of the bridges over the Danube arms and a completely new road through the floodplain to the northern river bank near the village of Jedlesee (Smital 1903; Slezak 1980).^13^ Human practices needed to be adapted to accommodate these changes. Probably in 1569, the *Tabor bridge* and the toll building were relocated to today’s *Augarten park*, approximately 800 m west of its former location (Fig. 2). Not only this bridge but all other bridges, including the *Wolf bridge* over the 800 m wide *Wolf arm*, had to be reconstructed. The moving of the *old Tabor* (*Tabor* I) to the *new Tabor* (*Tabor* II) that was described is an important turning point in Vienna’s history: the only land transport route ran via this point towards the north.^14^

In the following decades up to c. 1607, Viennese authorities attempted to dam up the newly formed main branch of the Danube and to force the waters into the old, “correct” river bed.^15^ The main goal of these efforts was to restore the city’s accessibility via the waterway (Fig. 3 in Hohensinner et al. 2013, in this issue).

Neither hydraulic engineering techniques nor financial resources allowed for effective measures (Thiel 1904, 1906). The considerable regulation efforts at the bifurcation of the former *Tabor arm* and the *Wolf arm* near Nußdorf in the sixteenth century have been interpreted in the literature as attempts to secure the flow and the navigability in a side arm, the *Wiener arm* and later *Donaukanal* (Mitis 1835; Thiel 1904; Mohilla and Michlmayr 1996; Altfahrt 2000). The plan, however, was much more ambitious: the Viennese tried to at least partly block the new main channel and to divert the main flow into the old river bed.

### Coming to terms with the new reality (1610–1683)

**Fig. 3:**
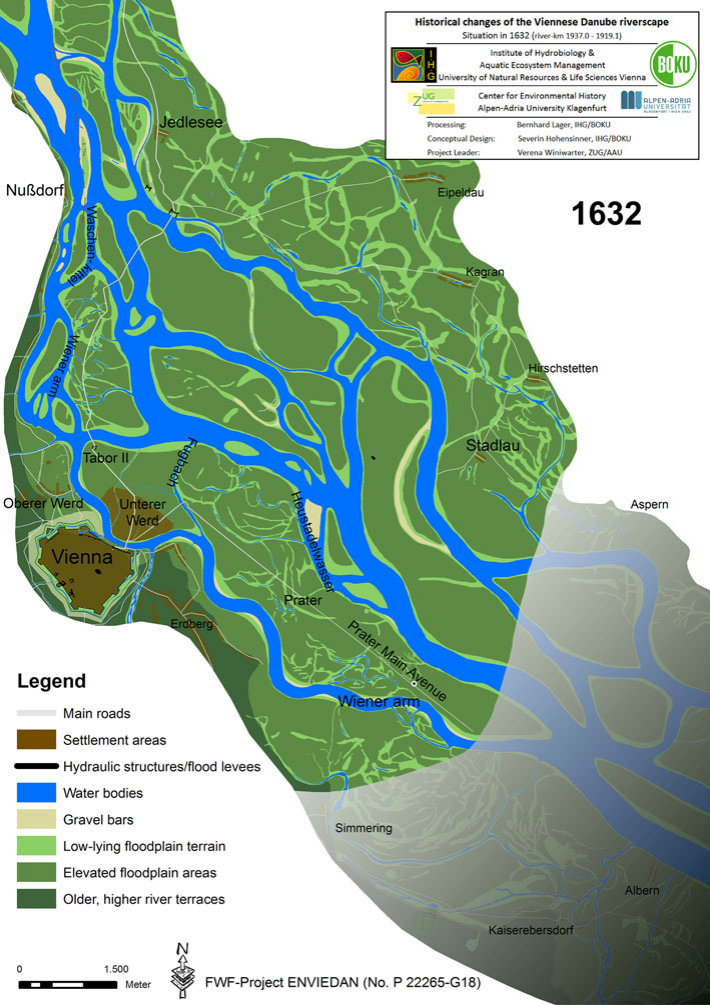
Reconstructed state of the Danube riverscape in Vienna in 1632

The reconstruction of the riverscape in 1632 reflects the ongoing terrestrialization process in the former main arm (*Tabor arm*; Fig. 3). As the main source, we used a map from 1632 that covers almost the entire study site. It was so far completely disregarded in the historical literature.^16^ In some parts, its geographical projection proved to be very inconsistent, potentially causing the incorrect positioning of some river arms in some floodplain areas without great importance for the interpretation of the overall historical state. Since the former main channel from c. 1570 is depicted on the map, it closes a gap in our knowledge about the development of the Danube between 1570 and 1632.

By 1632, the *Tabor arm* had developed into a side arm constituting the upper course of the *Wiener arm*. It was already very similar to the course shown in city maps after 1704. At the inflow near Nußdorf, two smaller arms had developed within the former wide river bed—the *Wiener arm* and the *Waschenkittel*.^17^ Several larger islands formed in the *Wiener arm* further downstream. They originated from the cut-off of the vertex of the *Tabor arm* between 1547 and 1565. Terrestrialization was additionally amplified by several tributaries that discharged to that arm, all carrying substantial sediment loads. The significantly reduced flow in the *Wiener arm* led to the formation of new terrain for the expansion of settlements close to the city centre, in the *Obere Werd* and *Untere Werd*.

After 1610, the Viennese authorities began to accept the new situation; the Danube was now further north and could not be moved. The available resources were now spent on maintaining a minimum flow to allow shipping in the remaining *Wiener arm*. In the meantime, the new main arm, the *Wolf arm*, had formed a distinct river bend to the south towards the city, where it reached the remnants of the former *Tabor arm*. Interestingly, the pre-eighteenth century history of the Viennese Danube is strongly focussed on the regulation works in the Danube near Nußdorf and in the *Wiener arm*. The vast remainder of the riverscape was thought to be untouched by river engineering measures prior to the mid-eighteenth century. In fact, amongst other measures, two cut-off channels up to 340 m long were excavated around 1649 several kilometres downstream of Nußdorf to the west and south of the village of Stadlau (Fig. 3).^18^ The cut-offs were constructed to protect the imperial hunting ground *Prater* from further erosion by a Danube arm later called *Heustadelwasser*. That river arm also eroded a longer section of the broadway *Prater Main Avenue* (Fig. 3 illustrates this situation several years prior to erosion). The cut-off channels could not mitigate the situation and three other cut-offs and additional measures were planned in response but obviously never implemented.

The reconstructions also yield new findings about a water body crucial for the urban development of the *Untere Werd* (*Leopoldstadt*) close to the city: *Fugbach* (or *Figgerl*), a side arm that constrained the settlement area to the east and separated it from the *Prater*. In 1570, the *Fugbach* did not yet connect the main arm with the *Wiener arm* as commonly described. Instead, it discharged into the later *Heustadelwasser* (Fig. 2).^19^ This means that the area later known as *Praterstern* was not cut off from the *Prater* and the *Prater Main Avenue* was directly accessible over the *Jägerzeile* (today’s *Praterstraße*) without crossing a larger water body. At least until 1572, waterborne transport of wood and other goods from the islands in the central riverscape to the city was only possible as far as to the Royal Bridge *(Khunigisches prugglein*).^20^ From there, carriages were needed for the further transport through the *Jägerzeile*. The transformation into the well-known state of the late seventeenth century took place between 1572 and 1632, when the *Fugbach* broke through the *Praterstern* area further south to the *Wiener arm* (Fig. 3). Since then, the direct access from the central riverscape via the *Fugbach* to Vienna significantly facilitated the transport.

During the following three decades until 1663, the *Wolf arm* increased its sinuosity and developed a new river bend whose vertex was only 1 km east from that of the *Tabor arm* 100 years earlier. This is precisely the river morphological situation with which the common history of the Danube in Vienna usually starts. Based on our reconstructions, tracing back the evolution of the main Danube arm in 1663 commonly referred to as *Fahnenstangenwasser*, the *Wolf arm* dating back to the early sixteenth century can be seen as its direct predecessor.

The Ottoman threat to the city in the decades that followed prevented more substantial hydraulic constructions directed at maintaining the navigability of the *Wiener arm*. Moreover, heavy disputes about the required financial funds between the emperor, the government and the estates (*Landstände*) of Lower Austria as well as differing opinions of the involved hydraulic engineers about the design of the planned water works led to further protractions (Thiel 1904). But one major effort was made between 1671 and 1680, when the imperial shipmaster Simon Peter Langsteger constructed a new spur dike at the inflow of the *Wiener arm* near Nußdorf, designed to guide the current into that arm (Thiel 1904, 1906).

### Intensifying the regulation efforts and planning the new city (1683–1760)

**Fig. 4:**
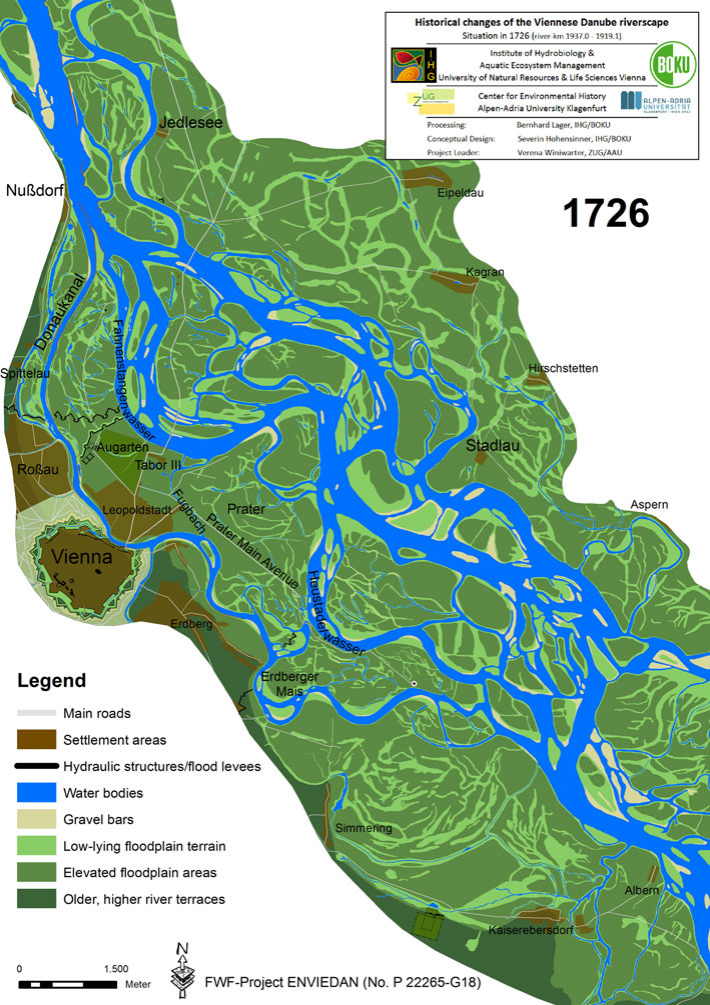
Reconstructed state of the Danube riverscape in Vienna in 1726

After the Ottoman siege, efforts were devoted to improving the precarious channel of supply via the *Wiener arm*. Parallel to planning a major expansion of Vienna’s fortifications, an attempt was made to finally resolve the unsatisfactory situation of the incessantly aggrading *Wiener arm* (Slezak 1980; Opll 1986, 2004).^21^ The situation had deteriorated despite the new spur dike at the inflow (1671–1680). Sediment input from the main arm and the tributaries had transformed the former broad *Tabor arm* to the more recent pattern of the upper *Wiener arm* with two smaller branches in its upper course. After controversial discussion, the decision was made to block the western branch of the *Wiener arm* along the hillslopes of the *Wienerwald* and to improve the flow capacity of the eastern arm, the *Waschenkittel*. Figure 4 shows the situation in 1726, two decades after the elaborate river engineering measures were completed.^22^

The first hydraulic works for the regulation project started c. 1686 with an elaborated spur dike (*Gegensporn*) at the northern Danube bank opposite Nußdorf. This was designed to deflect the current into the newly adapted inflow of the *Waschenkittel* (Thiel 1904, 1906; Fig. 4). Under the direction of Vizestatthalter Ferdinand Karl Graf von Welz, a straight canal, 1,140 m long, was excavated that connected the *Waschenkittel* arm to the *Wiener arm* further south. Until today, most of the literature views the excavation of the new channel as having been completed around 1598 by Freiherr Hoyos von Stixenstein (Baltzarek 1973; Czeike 1974; Mohilla and Michlmayr 1996).^23^ Based on the newly discovered map from Hoffmann von Anckherskron et al. (1700) and our reconstruction, we can specify the date more precisely as being between 1700 and 1703.^24^ Together with the new canal, Graf von Welz also constructed a very long guiding wall (*Teilungswerk*) at the new inflow. It was designed to lead the current into the canal. The old inflow of the *Wiener arm* was dammed up by 1704; the significantly modified *Wiener arm* has since then been called *Donaukanal*.

During this period, all bridges that had been destroyed during the siege in 1683 had to be restored.^25^ According to Slezak (1980), the bridges were first reconstructed at their old positions in 1690. The above-mentioned map from bridge-master Hoffmann von Anckherskron et al. (1700) allows a more precise localisation: The new bridge over the main Danube arm was reconstructed 90 m upstream from the old one, and the most northerly bridge (*Schwarzlackenbrücke*) was rebuilt about 430 m further upstream than before. In 1704, under the direction of Hoffmann von Anckherskron, who developed a revolutionary method for a faster bridge construction, the bridges were finally relocated further downstream to the positions they had until 1870 (Slezak 1980; see bridges in Fig. 4).^26^ The *Tabor* toll building was also relocated further downstream. It is now known that the *new Tabor* (*Tabor* III) was located only 300 m east of the *Tabor* I that had existed until 1565.

Despite all the efforts and the high costs (c. 400,000 gulden for hydraulic works alone), the *Donaukanal* was still not fully navigable all year round (Thiel 1904). Until 1712, Graf von Welz and various engineers tried to solve the problem by repeatedly lengthening the guiding wall and by constructing a groundsill (*Sohlschwelle*) across the whole Danube river bottom in order to divert the current into the *Donaukanal* (Slezak 1978). Upon completion of all these elaborate constructions, several problems became apparent: Nearly every year, the complex hydraulic constructions on the Danube had to be restored after ice jam floods, and the *Donaukanal* aggraded rapidly. In the following decades, the guiding wall at the inflow of the *Donaukanal* and the deflector construction at the opposite Danube bank were adapted several times. The goal was to direct as much water as possible from the Danube into the *Donaukanal* while controlling the volume to prevent the city from being flooded and to minimise ice drift into the channel. In the end, the navigability of the *Donaukanal* was only minimally improved. As a side arm of the Danube, the *Donaukanal* had a lower gradient and consequently lower flow velocity than the main channel. The sediments carried into the *Donaukanal* by the current of the main arm aggraded in its river bed. The more success the river authorities had with the diversion of water into the *Donaukanal*, the more material entered with the water and was deposited in the canal. Human practices of regulation had changed the arrangements of the river, initiating a spiral of new practices to deal with the consequences of the former ones, accordingly changing arrangements and so forth, and in the process completely transforming the socio-natural site of the Viennese Danube.

The excavation of the new canal between 1700 and 1703 resulted in altered flow conditions further downstream in the canal. Due to that, between 1704 and around 1712, large parts of the *Spittelau* floodplain were eroded and had to be protected with elaborate guide walls (Fig. 4).^27^ The new guide walls, in turn, deflected the current and amplified the erosion of terrain downstream at the left bank near the *Augarten park*, which gave rise to substantial hydraulic constructions throughout the eighteenth century (Thiel 1906).^28^ The precarious situation of the *Donaukanal* was additionally intensified by the sediments of several tributaries that were also partly deposited in the *Donaukanal* (Pasetti 1859).

Another important development for the Viennese riverscape occurred at the *Heustadelwasser*, where the Danube had eroded the *Prater Main Avenue* between 1632 and 1663. Hydraulic measures implemented in the seventeenth century in order to prevent further erosion of the *Prater* proved to be useless. The Danube shifted further south and, probably between 1715 and 1717, broke through to the lower *Donaukanal* (Fig. 4).^29^ In the medium term, this would have re-arranged the channel network downstream of Vienna. Consequently, a solid diversion dam was constructed before 1726, separating the *Heustadelwasser* from the *Donaukanal*.^30^ In addition, two meander loops of the *Donaukanal* located at that site were cut off in 1716 and 1726, respectively.^31^ This would be the most radical human intervention along the lower *Donaukanal* until 1832.

Prior to 1726, the river bend of the main Danube arm, the former *Wolf arm* now called *Fahnenstangenwasser*, again shifted slightly to the south. This eroded areas of the *Leopoldstadt* (the former *Unterer Werd*) that had aggraded in the river bed of the former *Tabor arm* after 1565. The last remnants of the *Tabor arm* were reduced to backwaters 10–15 m broad, located within the *Augarten park,* and the newly aggraded sites were integrated into the park or hosted the *Tabor* III from 1704 (Fig. 4). In order to prevent further channel migration and the erosion of the *Augarten* and the *Tabor*, the cut banks at these sites repeatedly had to be protected (Thiel 1904).^32^ The subsequent development of the main arm between 1726 and 1760 was characterised by a gradual retreat of the Danube from its river bend at the *Augarten park*, while the northern, straighter main arm gained in flow capacity. This process can be considered analogous to the abandonment of the sinuous *Tabor arm* around 1565, but change was slower than it had been 150–200 years earlier.

### Thinking about the big solution (1760–1850)

Around 1760, river experts began to consider a large-scale solution for the problematic navigation conditions and for providing better flood protection for the capital and the villages on the left bank of the Danube. An ambitious project proposed by Ingenieurs-Hauptmann Spallart in 1760 was not realised, while other proposals suffered the same fate (Thiel 1906).^33^ At that time, the frequency and intensity of floods were gradually increasing. Between 1768 and 1789, a total of 36 floods were documented, 7 of these being very severe (Fig. 7).^34^ Increased fluvial activity can be seen in the context of climatic changes towards the end of the *Little Ice Age*, volcanic eruptions in Iceland in 1783/84, and large-scale land use change in the drainage basin (Bork et al. 1998; Vasold 2004; Pfister and Brazdil 2006). The reconstructed state for 1780 reflects the reaction of the Danube to the altered hydrological conditions. The “First Military Survey” of 1780 was used as the main basis for reconstruction.^35^ Since its map projection shows great inconsistencies, we integrated information from several other maps and hydraulic construction plans with a higher level of detail and more accurate positioning.

**Fig. 5:**
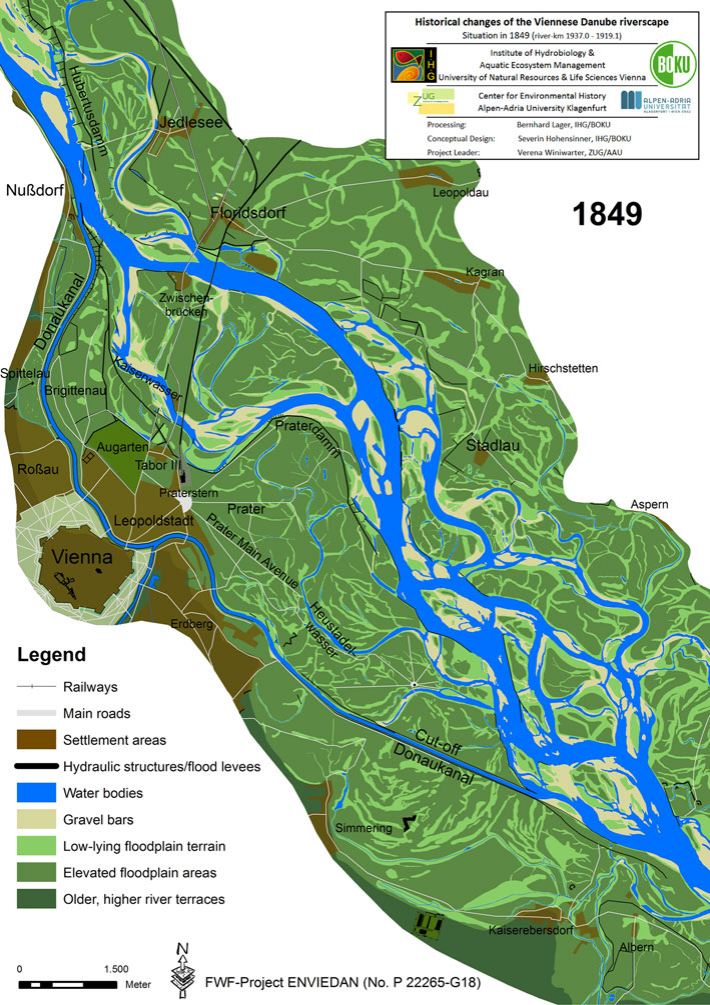
Reconstructed state of the Danube riverscape in Vienna in 1849 prior to the great regulation programme

Until 1780, the Danube gradually concentrated its flow in the northern arm, whose width increased, while new islands and gravel bars developed in the *Fahnenstangenwasser*, the southern sinuous arm close to the city. The northern arm, in turn, started to develop a new river bend towards the south and the river bed widened significantly up to 600 m due to the high fluvial activities between the 1760s and 1780s. Flooding culminated in 1786, a year with several very severe floods, and in 1787, with what was probably the second highest flood of the last 500 years, the so-called *Allerheiligengieß* (Pasetti et al. 1850; k.k. HZB 1908). The increasing flood threat gave rise to a series of hydraulic constructions. The *Fugbach*, the side arm that connected the *Fahnenstangenwasser* to the *Donaukanal* east from *Leopoldstadt*, was blocked in 1775 because it added to the flood threat. From 1779/80, a longer section of the remaining *Fugbach* was filled up and today’s *Praterstern* connecting the *Leopoldstadt* and the *Prater* was constructed (compare Figs. 4, 5; Bergenstamm 1812).^36^ In *Leopoldstadt*, a flood protection levee was installed between 1775 and 1783 along the *Fahnenstangenwasser* (now called *Kaiserwasser*) extending from *Augarten park* far into the *Prater* (Fig. 4). Despite a longer dispute between several hydraulic engineers, the newly established imperial *Navigationsdirection* 37 and other administrative institutions, Empress Maria Theresia entrusted the Hungarian engineer Johann Sigismund Hubert with the construction of the first systematic levee system in 1776/77 (Thiel 1906). It was intended to protect against flooding and to improve navigation conditions upstream of Vienna. Until 1784/86, a levee system almost 7 km long extending along the northern river bank from Langenzersdorf to opposite Nußdorf was erected (later called *Hubertusdamm*; Fig. 5).

The *Hubertusdamm* was partly destroyed soon after completion by the catastrophic *Allerheiligengieß* in 1787, prompting a discussion in which Joseph II was personally involved as to whether it was actually beneficial or would instead increase the flood risk (Thiel 1906). The dam was not rebuilt until 1849, when the dike was fortified and heightened (Pasetti 1859). In the 6 years following the *Allerheiligengieß*, an additional levee was constructed in the *Brigittenau* along the *Fahnenstangenwasser*. As a result, by 1793, all urban areas adjacent to the *Fahnenstangenwasser* were protected by dikes (Fig. 5). The flood threat posed by the *Donaukanal* remained, however. Almost all banks along its upper course had been protected by 1780. Despite intensive regulation efforts, fluvial dynamics in the *Donaukanal* amplified, which called for an intensification of regulation. The increased dynamics can be linked at least partly to the river engineering measures themselves. The erosive force of the *Donaukanal was* concentrated in those sections that were still unprotected. In addition, the increased frequency and intensity of ice jam floods at that time intensified fluvial dynamics. Despite all the regulation efforts described, the predominant part of the Viennese riverscape had not been directly affected by river engineering measures; except for land use-induced changes, it constituted a near-natural riverine system.

A combination of the Viennese cadastral maps produced between 1817 and 1825 and the “Lorenzo-Karte” surveyed between 1816 and 1817 provide an optimal basis to assess the situation in 1817.^38^ Both show the situation after termination of the last meander evolution phase. The series of high floods in the 1780 s had led to a significant widening of the main arm. Several years later, the Danube developed two distinct meander loops in the widened profile, which were abandoned around 1805. In 1817, these meander bends already showed substantial accretions and flow, once again concentrated in a comparably straight arm further north.^39^ Efforts continued to enhance terrestrialization in the *Fahnenstangenwasser* along settled areas (compare Figs. 4, 5).

The following decades were characterised by protracted discussions on the technical and financial feasibility of various regulation projects. In 1825 a planning competition for the regulation of the Danube and the construction of a stable bridge was announced based on a former concept of the head of the imperial building council (*Hofbaurat*) Joseph Schem(m)erl. However it didn’t result in anything suitable for execution (Mohilla and Michlmayr 1996). Meanwhile, the construction of embankments along the main river arms was continued without an underlying master plan. In 1832/33, a cut-off canal 2,500 m long was excavated at the *Donaukanal’s* confluence with the Danube to reduce the danger of ice jam floods (Pasetti 1859; Fig. 5).^40^ In 1849, most of the flood protection dikes built between 1775 and 1793 still existed and new ones were being built. The newly excavated bed of the lower *Donaukanal* did not mitigate inundations in the city, because sediment accretion directly downstream from the outflow of the *Donaukanal* in the Danube fostered the formation of ice jams (Fig. 5).

Therefore the main arm of the Danube was deflected to the outflow of the *Donaukanal* in 1849–1850. Aggraded material was to be eroded to prevent future ice jams at that location (Pasetti 1859). Several other river engineering measures were accomplished in order to provide work for the needy Viennese workforce (so-called *Notstandsbauten*; Thiel 1906). But no further major measures were yet executed. Instead, the implemented hydraulic structures were repaired and improved several times after floods. By 1849, c. 40 % of the main channel was protected and several side arms had been cut off. This suppressed dynamic fluvial processes in large portions of the Viennese riverscape, which still featured several lotic and lentic water bodies and the distinct terrain relief of a floodplain.

### Discussing and realising the great Danube regulation (1850–c. 1880)

In 1850, another attempt was made to solve the ongoing Danube question: a committee, the *Danube Regulation Commission*, was formed and charged with elaborating comprehensive planning principles and evaluating the advantages and disadvantages of different regulation options. Besides the commission, several individual (self-appointed) experts tried to gain public attention by publishing studies for the Danube regulation. The need to expand the city had by that time become conceivable, so planning was to take this into account (Pasetti et al. 1850; Donau-Regulirungs-Commission 1868). Improved transport on and across the Danube and flood protection for the entire city and the creation of sufficient space for a possible expansion were all to be figured into the plan. At the same time, another important project for Vienna’s urban development was launched: the demolition of the fortification all around the historical city centre between 1858 and 1863 gave way for the inner expansion of the city and for the construction of the *Ringstraß*e boulevard. But it was another several years (until 1869/1870) before an ambitious project for the regulation of the Danube would actually be approached. In the meantime, side arms were dammed, flood levees were heightened several times and embankments were undertaken in the main arm, but without any general plan. Once the great Danube regulation programme had started in 1869/70, it took only 5 years to create the new course of the river, extensive flood levees and new urban development areas along the river banks.

**Fig. 6:**
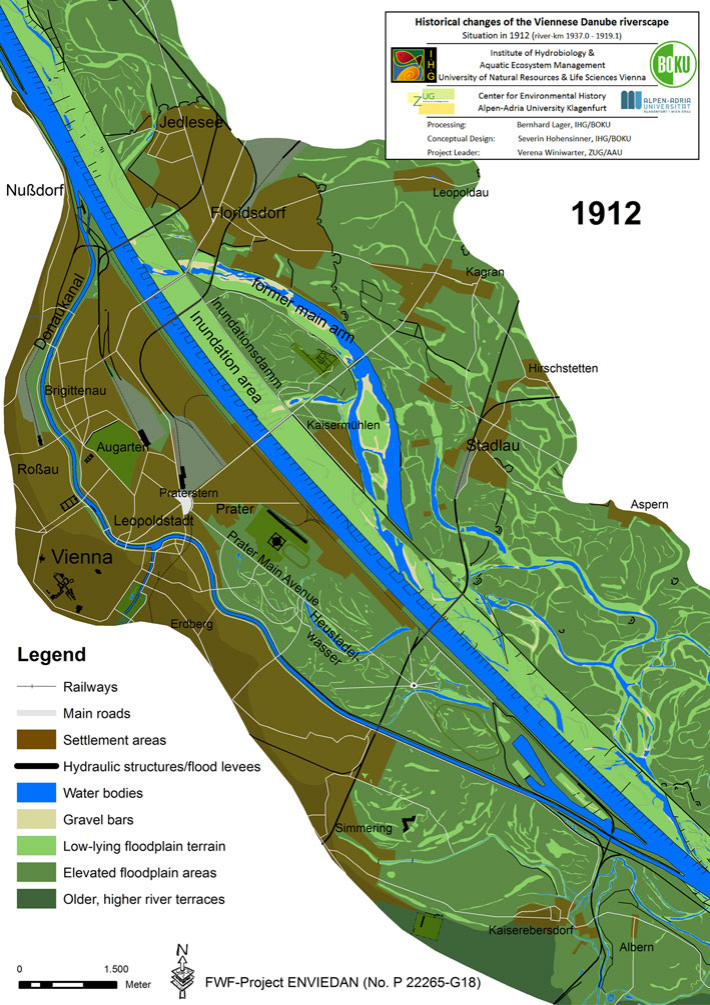
Reconstructed state of the Danube riverscape in Vienna in 1912, 37 years after completion of the great regulation programme

We reconstructed the state of the transformed riverscape directly after the first phase of the regulation programme in 1875 and 37 years thereafter in 1912 (Fig. 6). The primary source for the reconstruction of 1912 is the “Generalstadtplan” (“Municipal development plan”) from 1912 which was compiled based on detailed cadastral maps.^41^

What had happened? between 1870 and 1875, a new, straight bed for the Danube was created in order to prevent ice-jams, for which two cut-offs (6,638 and 2,845 m long) were excavated (Donau-Regulierungs-Commission 1898). Parallel to the new bed, a low-lying inundation area 470 m broad was excavated to enhance the discharge capacity of the bed during floods. The key feature of the system consisted of carefully constructed flood protection levees at both sides of the new Danube, designed to prevent the whole city from being inundated once and for all. The material from the upper cut-off (approx. 12.3 million m³) was used to fill up the *Kaiserwasser*, a side arm that originated in the sinuous *Fahnenstangenwasser* of the eighteenth century (compare Figs. 4, 5). Large parts of today’s districts of *Brigittenau* and *Kaisermühlen* were later erected on this material, creating compaction problems in the mid and long term. During the excavation works, about 163,000 m³ of older hydraulic structures, thousands of wooden piles and 18,400 running metres of sills and ties were removed, most of them from the location opposite Nußdorf where the deflecting spur dike system (*Gegensporn*) had been repeatedly reconstructed from the late sixteenth to the early nineteenth century (Lederer 1876; Prokesch 1876). The removal of the old hydraulic structures and the excavation of the new bed lowered the water table and consequently also the groundwater table in the surrounding areas by ca. 1.3 m (Wex 1876). This alleviated construction works on the newly created settlement areas because it reduced the costs for heightening the terrain. The *Donaukanal* was also expanded: almost 550,000 m³ of sediments were removed from its bed and used to heighten its banks and create new areas for settlement. In 1875, Viennese officials hoped that the centuries-old Danube issue had been finally solved. The *Donaukanal*, Vienna’s provisioning lifeline and therefore the main target of hydraulic engineering considerations, lost importance due to the changing technology: from now on, most of the Danube traffic was processed outside the city centre via steamships too large to pass the canal (see Gierlinger et al. 2013, in this issue).

### New threats (c. 1880–2010)

Cautious hydraulic experts argued that not all problems had been solved in the first years after the regulation. The dikes were deemed to be too low for very large floods and the river bed too wide, and thus too shallow for unobstructed navigability during low-flow situations. In the following decades, improvements were undertaken. These included partially heightening the dikes, constructing a new weir at the new inflow of the *Donaukanal* (1894–1899), and installing low water-control structures in the river bed (1898–1904; Fig. 6; Thiel 1906). A 30-year flood in 1897 and in particular a 100-year flood in 1899 showed that additional efforts were necessary to protect the city from being inundated (Waldvogel 1910/11). Sections of the flood levees would once again be repaired and heightened in the years to 1908.

The far-reaching river engineering measures in the late nineteenth century resulted in a complete transformation of the Viennese riverscape. While large areas along the new river bed were directly affected by the measures, more remote areas also experienced substantial changes, among others, intensive terrestrialization processes due to the reduced fluvial dynamics behind the dikes. After World War II, the flood protection facilities of the nineteenth century were considered unsuitable to protect the city. Further extensive river engineering measures between 1972 and 2010 were undertaken. A 21 km-long flood bypass called *Neue Donau* was built, flood levees were heightened and flood protection gates for harbours and the outflow of the *Donaukanal* were constructed, again transforming the riverscape in its entirety. Whether these measures will be sufficient to protect the urban agglomeration under altered climatic conditions remains unclear.

## Analysing the transformation from a technological-hydromorphological perspective

### Hydraulic engineering through the years

**Fig. 7:**
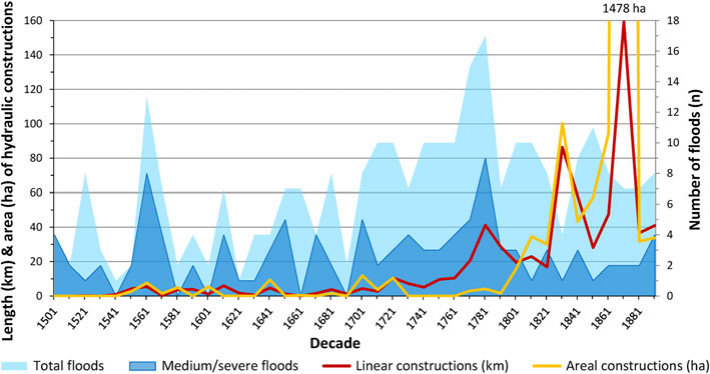
Historically documented linear hydraulic constructions (km/decade), areal river engineering measures (ha/decade) and number of floods per decade (*light blue* total documented floods, *dark blue* medium and severe floods). The values for the hydraulic constructions do not include measures at the Viennese Danube tributaries. In order to standardise the presented values, they have to be divided by the length of the centre axis of the valley floor (here: 26.5 km). The resulting values, km linear or ha areal measures per km valley length and per decade, can serve for comparison with other river sections

The long history of regulation of the Viennese Danube indicates the importance of riverine resources for the development of a city, in particular the use of the river as transportation route. It also underlines the importance of the social resources that must be mobilised to maintain regulation infrastructure. In the early sixteenth century, the main arm of the Danube River could still be used to navigate close to the city centre. The flow provided energy for transporting people and goods on the waterway. The tendency of the main arm to move north, away from the city, reduced its usability for transportation. The energy required to maintain and to improve transport increased. The hydraulic constructions reflected the technology and the economic resources available at the time of their creation. Large-scale solutions were not possible prior to the late eighteenth century; regulation efforts were concentrated at specific hot spots such as the inflow of the *Wiener arm* (*Donaukanal*) near Nußdorf. This piecemeal approach was consequential. In the sixteenth and seventeenth centuries, the hydraulic constructions fulfilled their functions poorly and were generally short-lived; their median functional life lasted only 3 years (half-value). An analysis of approximately one thousand eight hundred historically documented hydraulic measures shows that, on average at least c. 350 running metres of linear constructions (embankments, flood levees, spur dikes, guiding walls, etc.) had been accomplished annually between 1551 and 1600 (and potentially more that are not documented by the sources). Though historically documented more thoroughly, the workload in the seventeenth century declined to c. 220 running metres per year (Fig. 7).

The data reflect the amplified efforts in the late sixteenth century to counteract the increasing fluvial dynamics and to force the Danube into its former bed. The major hydraulic works in the seventeenth century, however, mostly aimed at merely keeping the *Wiener arm* navigable and the siege by the Ottoman army in 1683 interrupted these works for several years. The marked increase in flood activity together with the land requirements for urban growth in the second half of the eighteenth century is reflected by an annual total of c. 2,210 running metres of linear constructions while areal measures like cut-off channels or fillings of water bodies had rarely been implemented (see peak in 1781 in Fig. 7). At that time, major regulation works that were technically more sophisticated and more durable than their predecessors had been implemented; their median functional life increased to 10 years and on average to 17 years (arithmetic mean). The difference between the two values signifies that most constructions still were short-lived; only a few already functioned properly for longer periods. So the amount of energy necessary to maintain their functionality was still high: they had to be repaired (almost) annually. The costs of restoring those that had gone unattended for several years were even higher.

The focus of hydraulic measures until the early nineteenth century was on hot spots near Nußdorf and along the *Wiener arm/Donaukanal* close to the city centre. Though the hydraulic structures near Nußdorf had to be renewed on a regular basis they showed a surprising long-term continuity from the mid-sixteenth century (*Gegenschlacht* and *Neue Schlacht*) to 1870, when the older constructions were wiped out during the great Danube regulation. While the spur dike/guiding wall system at the inflow of the *Wiener arm* is documented in detail in the historical literature, the technically elaborate *Gegensporn* opposite Nußdorf is poorly documented. But the remnants of these constructions significantly influenced the hydraulic and morphological conditions several kilometres up- and downstream until 1870 (Wex 1871; Prokesch 1876; Lederer 1876). A second early hot spot that has been largely forgotten is the area south of Stadlau, where today’s *Heustadelwasser* eroded the *Prater Main Avenue* and eventually reached the *Wiener arm* near the *Erdberger Mais*. Substantial work was undertaken in this area from around 1640 to 1726.^42^

In the late nineteenth century it finally became possible to alter the riverscape profoundly and permanently by using energy inputs far exceeding those of previous centuries. The implementation of a great regulation scheme would technically have been possible several decades earlier; financial problems along with severe disputes amongst the entrusted experts and administrative difficulties prevented an earlier solution (Donau-Regulirungs-Commission 1868; Thiel 1906). During the nineteenth century as a whole, an average of 5,160 running metres of linear constructions and 19.2 ha of areal measures were implemented annually, most of them between 1848 and 1875. The newly applied techniques in river engineering are reflected by an increase in median duration of functionality, which means that half of the constructions functioned for at least 23 years without requiring any major rehabilitation. Some of the hydraulic structures fulfilled their functions over longer time spans, which is shown by the arithmetic average of 40 years.

### Modifications of the riverscape and fluvial dynamics

**Fig. 8:**
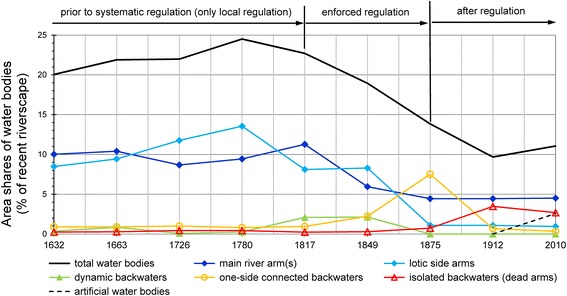
Composition of different types of water bodies in the Viennese riverscape, 1632–2010 (percentage area shares of the recent/post-glacial river-floodplain system)

We determined the areal extents of the various types of water bodies based on the historically mapped vegetation limits, which conform with the boundaries of the active channels (water bodies and unvegetated gravel/sand bars) and approximate summer mean water level (Osterkamp and Hedman 1982; Church 1992; Hohensinner et al. 2011). The analysis of the water bodies shows that river regulations prior to 1817 had little impact on the composition of the water bodies (Fig. 8). Between 1632 and 1817, lotic water bodies such as larger river arms and permanently flowed-through side arms would cover 18.5–23 % of the total recent (postglacial) riverscape. This means that 86–93 % of the overall water bodies were constituted by lotic river arms.^43^ Significant changes derived from climatic and ensuing hydrological changes. The increasing frequency and intensity of floods in the late eighteenth century is reflected in the reconstruction of 1780: at that time the Danube not only showed the largest total water-covered area within the time series, but also the highest share of lotic water bodies (with 23 % of the riverscape).

From 1817 onwards, water body composition gradually changed; main river arms declined and one-side connected backwaters increased (Fig. 8). During the great Danube regulation of 1870–1875, the Danube lost large parts of its former lotic arms, which were transformed into spacious backwater systems. The further development was characterised by intensive terrestrialization processes that had substantially reduced the size of the new backwaters by 1912. Overall, the transformation from the primarily lotic Danube system to today’s static riverscape is characterised by a quantitative reduction of water bodies and a qualitative change from lotic river arms towards lentic floodplain water bodies (dead arms).

**Fig. 9:**
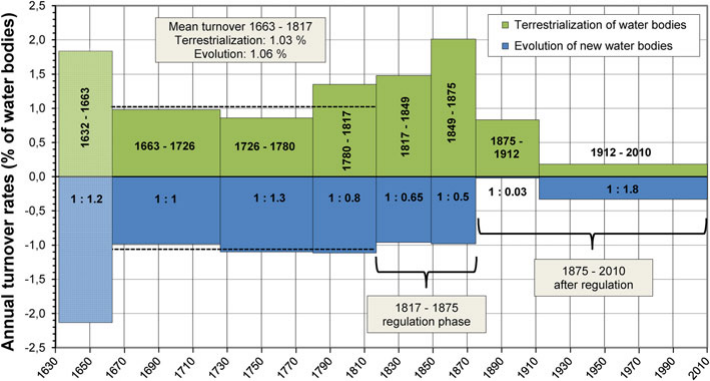
Intensity of fluvial dynamics in the Viennese riverscape, 1632–2010: evolution of new and terrestrialization of existing water bodies (percentage shares in relation to the respective area of the total water surface; the high values for the period 1632–1663 can be partly attributed to inaccuracies in the sources used for the reconstruction in 1632, but might also be due to increased fluvial turnover)

Our analysis of the evolution of new water bodies and the terrestrialization of old water bodies makes the altered fluvial dynamics visible (Fig. 9).

Prior to regulation, between 1663 and 1780, the formation of new river arms and terrestrialization were almost balanced; both affected about 1 % of the respective total area of the water surface. The progress of regulation between 1817 and 1875 is primarily reflected in increased terrestrialization processes, while the formation of new water bodies by and large remained stable. The effect of new hydraulic measures was counterbalanced by stronger river dynamics elsewhere in the system. Energy input by humans led to increased energy dissipation in other, insufficiently protected areas of the riverine system. Only when humans achieved the capacity to manipulate the total area of the Viennese Danube within a short time could the river’s energy be diverted into controlled paths and even used to support the regulation work (through erosive widening of narrow cut-off channels). In 1875, at the end of the great regulation project, the evolution of new river arms was largely blocked. Terrestrialization was the main morphological process in the Viennese riverscape between 1875 and 1912; after 1912 it gradually diminished (Fig. 9). The increase in new water bodies between 1912 and 2010 is due to the excavation of the flood bypass (*Neue Donau*) between 1972 and 1987 and to the excavation of new harbours.

## Synthesis

Literature on the Viennese Danube’s history has hitherto mainly highlighted two fundamental aspects: the retreat of the Danube from the historical city centre to the north since Roman times and the extensive efforts made to secure navigation in the precursor of today’s *Donaukanal* between the main Danube arm and the city. Both features can now be specified more precisely. Based on numerous historical sources providing spatial information, we can show that the Danube featured phases of both approach and retraction from the city centre within the last 550 years at the least. From around 1455–1565, a new, highly sinuous arm developed. It stretched several kilometres to the south, closer to the city. This was followed by a phase of natural channel straightening due to a river bend cut-off. The new main channel also developed a highly sinuous course until 1663/1700. The river would relocate its main current further north again thereafter. The last phase of river bend evolution lasted until around 1805, when a distinct double-meander loop had developed. Each phase of river bend/meander evolution and channel shift lasted ~100–130 years and over the long term, the average channel migration rate was c. 20 m per year. Our results also call for re-interpreting the centuries-long struggle to maintain navigability in the *Wiener arm/Donaukanal*. Until 1565, the upper course of the *Wiener arm* was, in fact, the main Danube arm and not a side channel. Regulation efforts in the sixteenth and early seventeenth centuries pursued a much more ambitious goal than merely regulating a side arm: they intended to deflect the main Danube arm into its former bed closer to the city, trying to counteract natural drivers such as channel dynamics.

Since most regulation efforts were concentrated around Nußdorf, upstream from Vienna, large parts of the Viennese riverscape were not directly affected by the measures undertaken up until the early nineteenth century. In the decades thereafter, the growing city called for a stabilisation of the dynamic riverscape in order to gain new settlement areas and to protect infrastructure in the floodplain, such as bridges, roads and railways. Though large parts of the riverscape were already stabilised in 1849 and therefore open for the city’s expansion, the flood threat remained unresolved. The systematic transformation of much of the Viennese riverscape into settlement areas was finally accomplished by the great Danube regulation programme of 1870–1875. The comprehensive regulation measures were meant to secure the future development potential of Vienna for centuries. Indeed, the current and future scope of action for urban expansion, transportation routes and sanitation—but also for remaining natural floodplain zones and urban open space—has been largely predetermined. The great regulation was undertaken on a river that had been substantially changed in preceding centuries, if not by the measures implemented then by their side-effects. Hydraulic and urban design considerations and courses of action were substantially influenced by the human-induced location of the *Donaukanal*, the former main arm of the river, now domesticated and channeled and relieved of its past as the Danube proper.

## Footnotes

1 WStLA, HA Urkunden, Sign. 3631.

2 Wien Museum, Topographische Sammlung, Sign. 48.068: Niclas Meldeman, "Der stadt Wien belegerung, wie die auff dem hohen sant Steffansthurn allenthalben gerings vm die gantze stadt zu wasser vnd landt mit allen dingen anzusehen gewest ist Vn von einem berumpten maeler…", 1530.

3 In this respect, our results contradict the hypothesis of a general relocation of the Danube away from the city as has been the view in nineteenth and twentieth century literature (Blumenbach 1834; Baltzarek 1973; Buchmann et al. 1984; Eigner and Schneider 2005).

4 Thiel (1904, 1906) provides a comprehensive review of historical documents on hydraulic measures undertaken regarding the Viennese Danube.

5 OeStA, AVA–FHKA, AHK, NOeHA, W 61/c/7/a (823), fol. 17r–23v.

6 OeStA, AVA–FHKA, AHK, NOeHA, W 61/c/7/a (823), fol. 20r,v.

7 OeStA, AVA–FHKA, AHK, NOeHA, N 27/b/1 (460), fol. 33r-34v, fol. 166r–168r.

8 The reconstruction is based on a number of maps, topographical views, descriptions of bridge locations and lengths, reports on hydraulic constructions and a historical survey from 1577 that allow an authoritative localization of riverine and human structures (e.g. Hans Mayr, "Warhafte Conterfactur der Stadt Wien" in Caspar Stainhofer 1566); Stiftsarchiv Klosterneuburg, Sign. Sp. 379: "Mappa über die umliegenden Dörfer bey Wien", 1632; OeStA, KA, HKR, Sign. Exped 1579: O. Waldegara, Longitudinal section of the Untere Werd, 1577; (see Hohensinner et al. 2013, in this issue).

9 e.g. OeStA, AVA–FHKA, AHK, NOeHA, N 27/b/1–3 (460–462); W 61/c/7/a and b (823, 824); W 61/c/87/a and b (875, 876); WStLA, Bürgerspital, Spitalmeisterrechnungen, Jg. 1548–1572.

10 OeStA, AVA–FHKA, AHK, NOeHA, W 61/c/87/a (875), fol. 57–59; OeStA, AVA–FHKA, AHK, NOeHA, W 61/c/b7/b (876), fol. 423–604.

11 OeStA, AVA–FHKA, AHK, NOeHA, W 61/c/7/a (823), fol. 257r,v, fol. 259r,v; Slezak (1980).

12 Compare total bridge length in Fig. 5 in Hohensinner et al. (2013), in this issue.

13 OeStA, AVA–FHKA, AHK, NOeHA, W 61/c/7/a (823), fol. 275–290, fol. 298–301.

14 In the historical literature of the last two centuries, this shift of the Tabor and the bridges has been largely ignored; in fact, Tabor II was generally referred to as Old Tabor (Bergenstamm 1812; Sekora 1948; Czeike 1981; Csendes and Opll 2001). Only Buchmann (1979) and Slezak (1980) mentioned Tabor I.

15 e.g. OeStA, AVA–FHKA, AHK, NOeHA, W 61/c/87/b (876), fol. 682ff.

16 Wien Museum, Topographische Sammlung, Sign. 95.961/4: "Detaillierte Darstellung der Wiener Donaulandschaft von 1632 mit Einzeichnung einer zwischen dem Wr. Bürgerspital und dem Stift Klosterneuburg strittigen Au sowie früheren Verläufen des Donauhauptstroms", 1632.

17 OeStA, AVA–FHKA, AHK, NOeHA, W 61/c/87/b (876), fol. 482–487; Moravian Library (Brno, Czech Republic), Mollova mapová sbírka, Sign. Moll-0000.397: Max Anton Hoffmann von Anckherskron, Jacob Hoffmann and Jacob Hermandt, "Disse Mappa ist von der Löbl. Kays. Wasserbaues Commission untern Prasidio des Hoch und Wohlgebohrnen Herrn Herrn Carl Ferdinand des Heyl. Röm: Reichs Graff und Herr von Welz …", 1700.

18 OeStA, AVA–FHKA, Kartensammlung, Sign. F 244: Thomas Claußnitz, "Wasser gebeus Abriß in der Donau zum brater gehörig. Ad acta", c. 1652.

19 Based on Wien Museum, Topographische Sammlung, Sign. 95.961/4: "Detaillierte Darstellung der Wiener Donaulandschaft von 1632 mit Einzeichnung einer zwischen dem Wr. Bürgerspital und dem Stift Klosterneuburg strittigen Au sowie früheren Verläufen des Donauhauptstroms", 1632, and two plans showing the morphological situation in the mid-sixteenth and the early seventeenth century, respectively (WStLA, Bürgerspital, Akten, Sign. XII/1).

20 WStLA, Bürgerspital, Spitalmeisterrechnungen, Jg. 1553–1572.

21 Compare fortification plans WStLA, Sign. 3.2.1.1.P1.221G: Herstal de la Tacle and J.P. v. Gehlen, (1697), and WStLA, Kartographische Sammlung, Sign. At 41: L. Anguissola and J.J. Marinoni, "Accuratissima Viennae Austriae Ichnographica Delineatio", 1706.

22 The main source for reconstruction is the so-called “Jagdatlas Kaiser Karls VI.” (“Atlas of imperial hunting grounds”) produced by J. J. Marinoni. Covering almost the whole study area, the map series is based on a geometrically correct survey and features a high level of detail (Marinoni 1751). OeNB, Kartensammlung, Sign. K I 98.480: J.J. Marinoni, "Neuer Atlas des Kayserl.en Wildban in Österreich unter der Ens", (1726–1729).

23 Except for Slezak (1978, 1980), who determined the construction phase based on numerous historical manuscripts from 1696 to 1703.

24 Moravian Library (Brno, Czech Republic), Mollova mapová sbírka, Sign. Moll-0000.397: Max Anton Hoffmann von Anckherskron et al. (1700).

25 It has generally been assumed that the bridges and the Tabor were relocated between 1688 and 1698, which probably reflects the lack of topographical sources known until now (Tschischka 1847; Alterthumsverein zu Wien 1911). Compare OeStA, KA, Kartensammlung, Sign. B IX b 106: Leander Anguissola, "Grundt Riss des Donau Strom von dem Dorff Höfflein bis auf Wienn… ", (1688), and WStLA, Kartographische Sammlung, Sign. At 41: L. Anguissola and J.J. Marinoni, "Accuratissima Viennae Austriae Ichnographica Delineatio", (1704/1706)..

26 OeStA, AVA–FHKA, AHK, NOeHA, W/61/c/7, fol. 835; Nö. Kammer, 522 rot, 30.12.1697.

27 Stiftsarchiv Klosterneuburg, Kartensammlung, Sign. Sp. 255 and Sp. 219: Graf v. Welz, Plans of the hydraulic constructions at the upper *Donaukanal*, c. 1706–1712.

28 National Library of Hungary, TK 369, Terkeptar, ST, 66: N.N., "Plan von dem Wiener Canal und denen darinnen bereits hergestellten mit rother Farbe bemerckten Werckern …", c. 1786.

29 Stiftsarchiv Klosterneuburg, Kartensammlung, Sign. Sp. 404: Joseph Haas, "Mappa Des Donau Stroms Sambt denen von selben ausgehenden Armen Und darinen befindlichen Häuffen wie auch der Tiefen des Stroms…", based on Vincenzo Coronelli, c. 1715; British Library, Sloane MS, Sign. 3603 ff: Vincenzo Coronelli, "Il Danubio Moderato Dalle Proposta segnate di Rosso del Padre Coronelli.", (1717).

30 OeNB, Kartensammlung, Sign. K I 98.480: J.J. Marinoni, "Neuer Atlas des Kayserl.en Wildban in Österreich unter der Ens", (1726–1729).

31 OeNB, Kartensammlung, Sign. K I 98.480: J.J. Marinoni, (1726–1729); OeStA, AVA–FHKA, Kartensammlung, F 528: Kollmann, "Grundh Rieß Von den durckh den Erthbergischen grundh durckh geschnidene Canal", (1716).

32 Wien Museum, Topographische Sammlung, Sign. 106.742: N.N., (1740).

33 OeNB, Kartensammlung, Sign. FKB A 21 4; OeStA, HHStA, Handschriften, Weiß 713, im Böhm. Katalog, Fol. 69a, Kodex 397.

34 Based on the analysis of historical Danube floods in the project ENVIEDAN and k.k. HZB (1908).

35 OeStA, KA, Kartensammlung, Sign. B IX a 242: "Josephinische Landesaufnahme", (1769–1785).

36 OeNB, Kartensammlung, Sign. FKB AA 10 1: M. Lauer, "Aufnahms-Plan Des Ober und Unteren Praters von Au-Garten bis zu dem Zusammenflus der Großen Donau…", (1779), and Sign. FKB 1340 C 13/1: M. Lauer, "Plan Des Au Gartens, der Ganzen Leopold Stadt, der Iäger Zeyl,…", (1780/81).

37 According to Thiel (1906), the *Navigationsdirection* was already founded in 1770; according to several other authors in 1773.

38 WStLA, Kartographische Sammlung, Sign. 2.2.3.2: "Franziszeischer Kataster", (1817–1825/30); NOeLB, Kartensammlung, Sign. B II 82: Christophorus de Lorenzo, "Nieder Oesterreichische Donau-Stromkarte", surveyed 1816–1817, published in 1819.

39 Based on NOeLB, Kartensammlung, Sign. B II 82: Christophorus de Lorenzo, (1819), and on GIS-reconstruction for 1817.

40 Figure 5 is based on the first altitudinal survey of the Viennese riverscape conducted by Valentin Streffleur in 1849 (Herrnegger 2007; Hohensinner et al. 2008). Technisches Museums Wien, Sign. L 20800: Valentin Streffleur and Carl Drobny, "Plastische Darstellung der Donau bei Wien nach der hydrotechnischen Vermessung vom Jahr (1849)", 1849; NOeLA, Regierungsarchiv, NOe Baudirection, Karton 494, Sign. Planschrank 10/Lade 7/III: Kazda and Nicolaus, "Lit. B: Plan des Donaudistrictes Wien", (1849–1850); Magistrate of Vienna, MA 29, Archive, without Sign.: K. Kilian, "Lage- u. Schichtenplan des Donaugeländes bei Wien (1849)", K. Kilian, 1970s?

41 WStLA, Kartographische Sammlung, Sign. 1701a: "Generalstadtplan", 1912.

42 OeStA, AVA–FHKA, Kartensammlung, Sign. F 244: Thomas Claußnitz, "Wasser gebeus Abriß in der Donau zum brater gehörig. Ad acta", c. 1652.

43 Lotic water bodies: flowing river arms; lentic water bodies: stagnant waters such as one-side connected backwaters or dead arms.
